# Impact of kidney biopsy on deciding when to initiate enzyme replacement therapy in children with Fabry disease

**DOI:** 10.1007/s00467-023-06050-5

**Published:** 2023-07-20

**Authors:** Jenny Avarappattu, Ariana Gaspert, Giuseppina Spartà, Marianne Rohrbach

**Affiliations:** 1 Department of Metabolic Medicine and Department of Nephrology, University Children’s Hospital Zurich, Zurich, Switzerland; 2 Department of Pathology and Molecular Pathology, University Hospital Zurich, Zurich, Switzerland

**Keywords:** Fabry Disease, Enzyme replacement therapy, Kidney biopsy, Podocyte, Children

## Abstract

**Background:**

Recommendations on when to start enzyme replacement therapy (ERT) in children with Fabry disease (FD) differ between guidelines. In this study, kidney biopsies of a cohort of 14 untreated children and one treated child were analyzed for their morphologic changes to determine whether early initiation of ERT is indicated.

**Methods:**

All pediatric FD patients (< 18 years old) diagnosed between 2003 and 2021 in our department who received a kidney biopsy were enrolled. Clinical symptoms; laboratory parameters regarding kidney function, such as eGFR, plasma urea, protein-creatinine, and albumin/creatinine ratio; and 14 kidney biopsies prior to ERT and one under treatment were retrospectively analyzed.

**Results:**

A total of 14 patients were enrolled, including 9 male and 5 female children, aged 3–18 years (median age 11). Seven of the enrolled children were 10 years old or younger. Histological analysis of kidney biopsy samples revealed severe vacuolization and accumulation of inclusions in podocytes and renal tubules. The majority of cases had no FD-specific clinical or laboratory features independent of age, gender, or genotype. The youngest FD patient presenting with isolated abnormal kidney biopsy was 3 years old.

**Conclusions:**

We demonstrate that histological lesions, typical for FD, can be observed in kidney biopsies at a very young age in patients without classical clinical symptoms or laboratory abnormalities. Thus, we recommend kidney biopsies as a possible tool for early diagnosis of renal involvement in FD. As a consequence of these early biopsy findings without a clinical correlate, an early initiation of ERT should be considered.

**Graphical abstract:**

**Figure:**
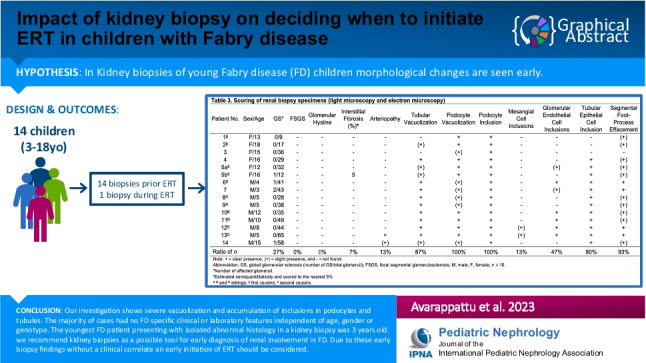
A higher resolution version of the Graphical abstract is available as Supplementary information

**Supplementary information:**

The online version contains supplementary material available at 10.1007/s00467-023-06050-5.

## Introduction

Fabry disease (FD) is a chronic, progressive, multisystem, X-linked lysosomal disorder caused by pathogenic variants in the galactosidase alpha (*GLA*) gene leading to a reduced activity of the enzyme galactosidase A (GALA) [1–3]. This mainly leads to a globotriaosylceramide (Gb3) accumulation within lysosomes in different organs [1–3], and less commonly to an accumulation of lipids such as digalactosyl ceramide and blood group B, B1, and P1 glycolipids [1–3]. The accumulation of Gb3 which begins in utero in the kidney cells is observed in adults mainly in the kidneys, heart, and cerebrovascular system and leads to severe organ complications, typically during the third to fifth decade of life [4–7].

FD can be categorized into two major forms: the severe classical form, with little residual enzyme activity, and the milder nonclassical form, also called late-onset phenotype [4]. Both forms affect female and male patients. Men with classical FD have a much higher risk of presenting with an event, especially they have a poorer kidney function and a higher left ventricular mass than women with classical FD and both women and men with non-classical FD [4]. Women rather show fibrosis in the absence of cardiac hypertrophy. Late-onset patients are mostly free of clinical symptoms in childhood and adolescence. They show a more variable disease course and are less severely affected; often, only one organ is affected by the disease [4]. Women with non-classical FD show the mildest disease course. For classical FD, there is a wide spectrum of disease severity and age of onset. Data on pediatric patients with classical FD show characteristic clinical features in male and female children, such as neuropathic pain, acroparesthesia, gastrointestinal symptoms, hypohidrosis, angiokeratomas, and ocular abnormalities such as cornea verticillate [2, 5, 8]. According to the Fabry Registry, the median age of onset of clinical symptoms is 6 years in males and 9 years in females [5]. However, accumulation of disease-specific deposits, such as podocyte inclusions in the glomeruli, may appear long before the patients present with typical clinical symptoms and organ-specific laboratory abnormalities [1, 2, 8, 9]. Initial signs are typically unspecific and may result in a significant delay of diagnosis [5]. This is also reflected by the fact that the majority of pediatric FD patients are detected by genetic screening due to a positive family history for FD [5]. Only a minority of pediatric FD patients are diagnosed as index patients [5]. Timely treatment is often hampered by a diagnostic delay leading to progressive damage of the kidneys, heart, and central nervous system with associated complications and thereby contributes to an increased morbidity and early mortality [2, 3, 10].

Enzyme replacement therapy (ERT) for FD was approved by the European Medicines Agency (EMA) for agalsidase beta (Fabryzyme®) and agalsidase alfa (Replage®) and by the U. S. Food and Drug Administration (FDA) for agalsidase beta (Fabrazyme®) in 2001 [11–13]. Several studies in adults have shown the positive effects of ERT, including reduction of glycolipid storage in different organs, reduced pain, and improvement of peripheral nerve function and sweating, as well as a reduction of cardiac hypertrophy [1, 8]. Clinical trials addressing the efficacy were all conducted in adults and only a small number of reports have documented the effects in children and adolescents [8]. In general, the treatment leads to an improvement of disease-related symptoms whereby a non-significant decrease of gastrointestinal symptoms was seen in boys [8]. In addition, it is believed that both agalsidase beta and agalsidase alfa have better clinical benefits when treatment initiation is established before Gb3 accumulation has induced kidney damage [3, 5, 14–17].

Morphological changes in the kidneys result from Gb3 accumulation in nearly all kidney cell types, with particularly dense accumulations found in podocytes and distal tubular epithelial cells [18]. They trigger interstitial inflammation and fibrosis, which can lead to chronic kidney disease (CKD) in adults [16, 17]. In a study with 58 patients (age > 16 years), accumulation of Gb3 in kidney tissue varied strongly but could significantly be reduced after 11 months on ERT [18]. ERT efficacy is less pronounced when the disease has reached the stage of CKD [1, 18–20]. In contrast, patients without impaired kidney function (no proteinuria and/or decreased glomerular filtration rate) are reported to have a better response to ERT [19]. In consequence, the current treatment recommendations for adults propose an early initiation of ERT to stop disease progression or even reverse organ damage [7, 21]. Nevertheless, the debate on when to initiate ERT in children is still ongoing and various recommendations have been made including different criteria regarding when to start ERT [3, 20].

Consensus criteria based on expert opinions include clinical symptoms and kidney involvement, such as proteinuria and/or elevated serum creatinine. However, morphological changes in kidney biopsies may be observed preceding proteinuria and/or elevated serum creatinine [2, 19, 22]. Yet screening for morphological changes in the glomeruli, as a marker for disease progression, has not been implemented as a standard diagnostic tool for FD prior to ERT. The absence of proteinuria/albuminuria is currently erroneously considered a measure to determine whether the kidney is affected or not. Therefore, the decision to start a treatment could be delayed in children who might benefit from initiation of ERT at an earlier time point. Thus, the decision to start ERT should not be based solely on proteinuria as a marker of kidney involvement [3].

Current US [20], French [23], and international [24] guidelines suggest different ages to start ERT (ranging from age 8 to 16). According to the latest publication in 2019 by Germain et al. [3], initiation of ERT is recommended at an age of 7 years independent of clinical or laboratory manifestations.

However, only little is known about the natural history of glomerular disease progression in the early phases of the disease or the silent kidney involvement in FD and is reviewed in Levstek et al. [21]. The study of Tøndel et al. is of particular interest, since progressive glomerular and vascular involvement in kidney biopsies from children and adolescents (age range 7–17 years) with FD could be observed even in the absence of proteinuria. Podocyte foot process effacement was found to be an early marker of nephropathy [7, 14]. Subsequently, the same author proposed that kidney biopsies are not only essential in the early diagnosis of nephropathy but might also serve in the evaluation of the response to ERT of early Fabry nephropathy [7]. Early initiation of ERT in children was documented to prevent progression of nephropathy on sequential kidney biopsies [14].

The intention of our study was to further investigate the kidney pathology by analyzing the kidney biopsies from a cohort of 14 untreated and one treated child with FD, as well as to review the evidence for an early intervention with ERT based on findings. Our results indicate that ERT initiation should be generally considered at younger ages, especially in male classical FD patients, but also in females with glomerular phenotypes, before FD-associated morphological changes in the kidney have developed.

## Methods

### Patients

All pediatric FD patients (< 18 years old) followed at the University Children’s Hospital Zurich diagnosed between 2003 and 2021 who underwent kidney biopsies were enrolled. All children, except two, had either albuminuria or microhematuria which led to the decision of performing a kidney biopsy. In patient 3, the kidney biopsy was performed due to clinical symptoms and in patient 6 due to family history with known kidney involvement.

Routine clinical monitoring included clinical visits every 6 to 12 months, measurement of kidney markers every 6 months, ECG and echocardiography every 12 months, and cardiac MRI every 5 years. FD was either suspected due to typical clinical symptoms and/or positive family history and was confirmed by the measurement of GALA activity in leukocytes, dried blood spots and/or plasma, and/or molecular genetics of the *GLA* gene. LysoGb3 measurement was included since 2019.

All data was collected retrospectively from the patient files and summarized in an Excel file. Kidney biopsy results were compared with biochemical measurements in urine and blood samples performed within the last 2 months before the kidney biopsy. Descriptive statistics were used to summarize the data. Due to the small number of patients a statistical analysis was not possible. The study was reviewed and approved by the local IRB (University Zurich). All participants signed a general consent to be included in research projects.

### Clinical symptoms

The clinical characteristics of the children, as shown in Table 1, were assessed using the Mainzer Severity Score Index [25]. Ophthalmologic and cardiac changes were evaluated by an ophthalmologist and a cardiologist, respectively. We included general characteristics like gender, age, and *GLA* sequence variant, and the most common clinical parameters: neurological involvement (acroparesthesia, tinnitus, dizziness, hypo- or anhidrosis), gastrointestinal symptoms (abdominal pain, diarrhea, constipation), ophthalmologic abnormalities (cornea verticillate), cardiovascular abnormalities, and angiokeratomas.

**Table 1:** General and clinical features at the time of the kidney biopsy

Patient no	Sex/age	Identification	Mutation	Enzyme activity	Acroparesthesia	Gastrointestinal symptoms	Eye changes	Hypohydrosis	Angiokeratomas	Cardiac changes	Tinnitus	Dizziness
1^β^	F/13	FS	c.744–745 del TA fs TER 254	Not done	+	+	+	−	−	−	−	−
2^β^	F/18	FS	c.744–745 del TA fs TER 254	0.32 mE/mg Protein	( +)	+	−	−	−	−	−	−
3	F/15	FS	c.1168insT	0.08 mE/mg Protein	+	+	−	−	+	−	−	−
4	F/16	FS	IVS1 + 1605_IVS2 + 618	0.07 mE/mg Protein	+	+	−	−	−	−	−	−
5a^δ^	F/12	FS	c.1033 T > C (S345P)	16.5 mmol/h/mg	−	−	−	−	−	−	−	−
5b^δ^	F/16	FS	c.1033 T > C (S345P)	31.9 mmol/h/mg	−	−	−	−	−	−	−	−
6^δ^	M/4	FS	c.1033 T > C (S345P)	Not done	−	−	−	−	−	−	−	−
7	M/3	FS	M42T	2.3 mmol/h/mg	+	+	−	−	−	−	−	−
8^A^	M/5	FS	p.C52R	Not done	( +)	−	−	+	−	−	−	−
9^A^	M/5	FS	p.C52R	Not done	−	+	−	+	−	−	−	−
10^B^	M/12	FS	c581C > T, p.T194l	Not done	+	+	−	+	−	−	−	−
11^B^	M/10	FS	c581C > T, p.T194l	Not done	+	+	+	+	−	−	−	−
12^D^	M/8	FS	c.901C > T	0 mmol/h/mg	( +)	−	−	−	−	−	−	−
13^D^	M/5	FS	c.901C > T	0 mmol/h/mg	−	( +)	−	−	−	−	−	−
14	M/15	FS	c.905G > T	1.8 mmol/h/mg	−	−	−	−	−	+	−	−
Ratio of *n*					60%	60%	13%	27%	7%	7%	0%	0%

*M*, male; *F*, female; *FS*, family screening; *n* = 15

^A^ ^B^ and ^D^ siblings; ^β^ first cousins; ^δ^ second cousins

Note: +  = clear presence, ( +) = slight presence, and −  = not found

Enzyme level reference range: 0.12–0.56 mE/mg Protein l 19.2–44.4 mmol/h/mg

### Kidney-associated laboratory features

The following kidney-specific parameters were included: estimated glomerular filtration rate (eGFR) was expressed in millimeters per minute per 1.73 m^2^ according to the Schwartz formula [26] using the local factor *κ* of 40 for all children based on the plasma creatinine, which was measured by an enzymatic method. Other kidney-related parameters include plasma urea, protein/creatinine, and albumin/creatinine ratio, which were calculated from spot urine by measuring protein and albumin content (by immunoturbidimetric assay). Impaired eGFR was defined as < 90 ml/min per 1.72 m^2^. Elevated plasma urea was defined at > 7 mmol/L, microalbuminuria with an albumin/creatinine ratio > 3.4 mg/mmol Crea, and proteinuria with a protein/creatinine ratio > 20 mg/mmol Crea. Nephrotic range proteinuria is defined as a protein/creatinine ratio > 200 mg/mmol Crea. Urine sediment was analyzed by the flow cytometric method.

### Morphological characteristics of the kidney

Since 2011, the University Children’s Hospital Zurich routinely performed kidney biopsies in all pediatric FD patients to evaluate the degree of kidney damage and confirm the indication for ERT.

Based upon the studies of Tøndel et al. [2] and the American [20] and French [3] recommendation for diagnosis, management, and treatment of FD, we decided to perform a baseline kidney biopsy prior to ERT, to also confirm the diagnosis, at the age 4–6 for boys and 12–15 for girls; a second biopsy is usually planned after 5 years on ERT or as a follow-up after 5 years, when the patient was not started on ERT. Within the period investigated in this publication, only one patient had a follow-up biopsy. All kidney biopsies were performed by an experienced nephrologist. The morphological characteristics were analyzed on light and electron microscopy by a specialized pathologist. To compare our results with current studies, one nephropathologist (A.G.) scored the biopsy samples based on a modified scoring system from Tøndel et al. [14]. Global glomerular sclerosis, focal segmental glomerulosclerosis, glomerular hyaline, interstitial fibrosis and arteriopathy, with PAS-positive hyaline-like material in the media of arterioles or arteries, replacing smooth muscle cells, was defined as Fabry arteriopathy [27]. Podocyte and tubular vacuolization were assessed on hematoxylin and eosin-stained and periodic acid-Schiff-stained slides. Inclusions in podocytes, mesangial cells, and tubular epithelial cells were assessed on methylene blue-azure stained semi-thin sections and with electron microscopy. Podocyte foot process effacement was evaluated on electron microscopy images and scored as segmental if comprising less than 50% of the capillary loop circumference. Plus signs indicate a clear occurrence of the feature, plus signs in parentheses indicate a slight occurrence, and minus signs indicate the absence of scored feature [14].

### Treatment with ERT

The EMA approved agalsidase alfa and beta for ERT in FD in 2001. Pediatric patients at the University Children’s Hospital Zurich are currently treated with agalsidase alfa or agalsidase beta. Depending on the age of the patient this treatment is stated as off label, specifically under the age of 7.

## Results

### Fabry cohort, clinical and laboratory findings

A total of 14 patients were enrolled, including 9 male and 5 female children. One individual (patient 5, Tables 1 and 2) had two consecutive kidney biopsies, one before (5a) and one on ERT (5b). A total of 15 kidney biopsies were investigated. All patients were identified based on family history, respectively family screening. FD was confirmed using biochemical and/or genetic testing. All individuals were classified as having classical FD. Kidney biopsies prior to ERT were performed in a total of 14 patients with a median age of 11. 7 of the enrolled children were 10 years old or younger. Multiple affected individuals from 5 families were included: patients 1 and 2 are first cousins, 5 and 6 s cousins, and 8 and 9 twins. Children 10 and 11 and 12 and 13 are siblings.

**Table 2:** Laboratory data at the time of the kidney biopsy

Patient no	Sex/age	Serum creatinine (µmol/L)	Urea (mmol/L)	eGFR (mL/min/1.73 m^2^)	Protein/creatinine (mg/mmol)	Albumin/creatinine (mg/mmol)	Urine sediment	Urine status
1^β^	F/13	58	3.2	99	ns	1.3	Normal	Normal
2^β^	F/18	51	5.3	120	13	6.14	Normal	Normal
3	F/15	42	3.4	148	ns	ns	Normal	Normal
4	F/16	56	4	127	ns	5.7	Normal	Normal
5a^δ^	F/12	45	3.5	131	11	6.37	Erythrocytes: 17/µlº	Blood: + +
5b^δ^	F/16	50	4.3	123	104	70	Normal	Protein: + +
6^δ^	M/4	32	5.3	122	ns	ns	Normal	Normal
7	M/3	< 27	5.3	144	15	0.8	Normal	Normal
8^A^	M/5	30	5.3	148	ns	2.5	Normal	Normal
9^A^	M/5	27	5.3	164	ns	0.8	Normal	Normal
10^B^	M/12	47	5.3	113	10	0.9	Normal	Normal
11^B^	M/10	43	5.3	123	ns	1.9	Normal	Normal
12^D^	M/8	54	5.3	86	ns	1.5	Normal	Normal
13^D^	M/5	43	5.3	100	11	1.6	Normal	Normal
14	M/15	69	5.1	114	7	0.4	Normal	Normal

*eGFR*, estimated glomerular filtration rate (Schwartz formula (26)); *ns*, nothing special; *M*, male; *F*, female

^A^ ^B^ and ^D^ siblings; ^β^ first cousins; ^δ^ second cousins

ºNormal-cut-off flow cytometry < 12/µl

### Kidney biopsy findings

The morphologic changes in the kidney biopsy specimens (light and electron microscopy) for all our patients are shown in Table 3. All morphological changes are typical for FD. Only chloroquine toxicity would show similar changes in kidney biopsies. However, there was no known chloroquine intake in our patients.

**Table 3:** Scoring of kidney biopsy specimens (light microscopy and electron microscopy)

Patient no	Sex/age	GS*	FSGS	Glomerular hyaline	Interstitial fibrosis (%)º	Arteriopathy	Tubular vacuolization	Podocyte vacuolization	Podocyte inclusion	Mesangial cell inclusions	Glomerular endothelial cell inclusions	Tubular epithelial cell inclusion	Segmental foot process effacement
1^β^	F/13	0/9	−	−	−	−	−	+	+	−	−	−	( +)
2^β^	F/18	0/17	−	−	−	−	( +)	+	+	−	−	−	( +)
3	F/15	0/36	−	−	−	−	-	( +)	+	−	−	−	−
4	F/16	0/29	−	−	−	−	+	+	+	−	−	+	( +)
5a^δ^	F/12	0/32	−	−	−	−	( +)	+	+	−	( +)	+	( +)
5b^δ^	F/16	1/12	−	−	5	−	( +)	+	+	−	−	+	( +)
6^δ^	M/4	1/41	−	−	−	−	+	( +)	+	−	+	+	+
7	M/3	2/43	−	−	−	−	+	( +)	+	−	( +)	+	+
8^A^	M/5	0/28	−	−	−	−	+	( +)	+	−	−	+	( +)
9^A^	M/5	0/38	−	−	−	−	+	( +)	+	−	−	+	( +)
10^B^	M/12	0/35	−	−	−	−	+	+	+	−	+	+	( +)
11^B^	M/10	0/49	−	−	−	−	+	+	+	−	+	+	( +)
12^D^	M/8	0/44	−	−	−	−	+	+	+	( +)	+	+	+
13^D^	M/5	0/65	−	−	−	+	+	+	+	( +)	+	+	+
14	M/15	1/58	−	−	−	( +)	( +)	( +)	+	−	−	+	( +)
Ratio of *n*		27%	0%	0%	7%	13%	87%	100%	100%	13%	47%	80%	93%

+  = clear presence, ( +) = slight presence, and −  = not found

Abbreviation: *GS*, global glomerular sclerosis (number of GS/total glomeruli); *FSGS*, focal segmental glomerulosclerosis; *M*, male; *F*, female; *n* = 15

*Number of affected glomeruli

ºEstimated semiquantititaticely and scored to the nearest 5%

^A^ ^B^ and ^D^ siblings; ^β^ first cousins; ^δ^ second cousins

Light microscopy showed podocyte vacuolization in all patients. In 13 out of 15 samples, tubular vacuolization was observed. Glomerulosclerosis, focal minimal interstitial fibrosis, and arteriolopathy were rare findings. Global glomerulosclerosis was found in 3 out of 14 patients whereby patient 7 presented an increased number of globally sclerosed glomeruli (2/43) for his age. Focal minimal interstitial fibrosis was seen in one patient. Fabry arteriopathy in the form of arteriolar lesions with hyaline-like material replacing smooth muscle cells in arterioles occurred in 2 patients. Podocyte inclusions documented by electron microscopy were present in all patients.

All patients, except for one 15-year-old girl (patient 3) with isolated podocyte vacuolization and inclusions without clinical or laboratory features of kidney involvement, presented mild segmental podocyte foot process effacement, comprising less than 50% of the capillary loop circumference. Our investigations revealed that morphological kidney changes are not always associated with clinical and laboratory findings. One child (patient 13), with 8 out of 12 positive light and electron microscopic criteria, solely complained about rare abdominal pain and showed no laboratory abnormalities. On the other hand, while patient 3 showed distinctive clinical features and angiokeratoma, the histology in this case only showed podocyte vacuolization and inclusions. In addition, children without any evident clinical symptoms can manifest severe morphological changes in their biopsies (patient 5a, 5b, and 6).

In one patient (patient 5b), on treatment with agalsidase alfa for 4 years, the follow-up kidney biopsy revealed a complete disappearance of glomerular endothelial cell inclusions. However, an onset of mild interstitial fibrosis and glomerulosclerosis was observed.

To summarize, there was severe vacuolization and accumulation of inclusions in podocytes in all patients, in renal tubules in 13 out of 14 patients, and a combination with segmental foot process effacement was seen in 13 out of 14 patients. Fabry arteriopathy was found in two patients.

## Discussion

This study investigates the kidney morphological changes found through biopsies in 15 children with FD as young as 3 years to extend the current data, in particular with focus on the clinical phenotype and initiation of ERT. Earlier studies by Tøndel et al. [2] investigated kidney biopsy findings in children and adolescents with FD in a smaller cohort; all but one were older than 10 years old. Two additional studies analyzing kidney biopsy findings in pediatric FD focused either on foot process effacement [7] or on the correlation between clinical parameters, LysoGb3, podocyturia, and kidney biopsy findings [28].

ERT for FD has been approved by the FDA and the EMA in 2001. Previously published recommendations suggest a treatment initiation as soon as patients present with FD-specific symptoms regardless of age or sex [20]. However, clear guidelines for pediatric FD regarding the appropriate age at which to start ERT are vague. Data on kidney biopsies investigating response to ERT as well as histological evidence of Gb3 accumulation in asymptomatic boys with classical FD suggested to consider beginning ERT around the age of 8–10 years [20, 29]. For asymptomatic girls with FD-causing mutations, primary clinical follow-up is recommended [3, 20, 23, 24]. It is believed that ERT should only be initiated when organs, such as the kidneys, are involved; however, organ screening through kidney biopsies is rarely performed.

Our study evaluated the early diagnosis of Fabry nephropathy in biopsies in correlation to ERT start, applying a semiquantitative scoring, based on a slightly modified version of the scoring protocol published by Tøndel et al. [14]. In contrast to Najafian et al. [30], who applied unbiased stereological quantitative methods to electron microscopic changes of Fabry nephropathy to determine the relationship between parameters of glomerular structure and kidney function, we did not intend to correlate our findings with kidney function.

**Fig. 1:**
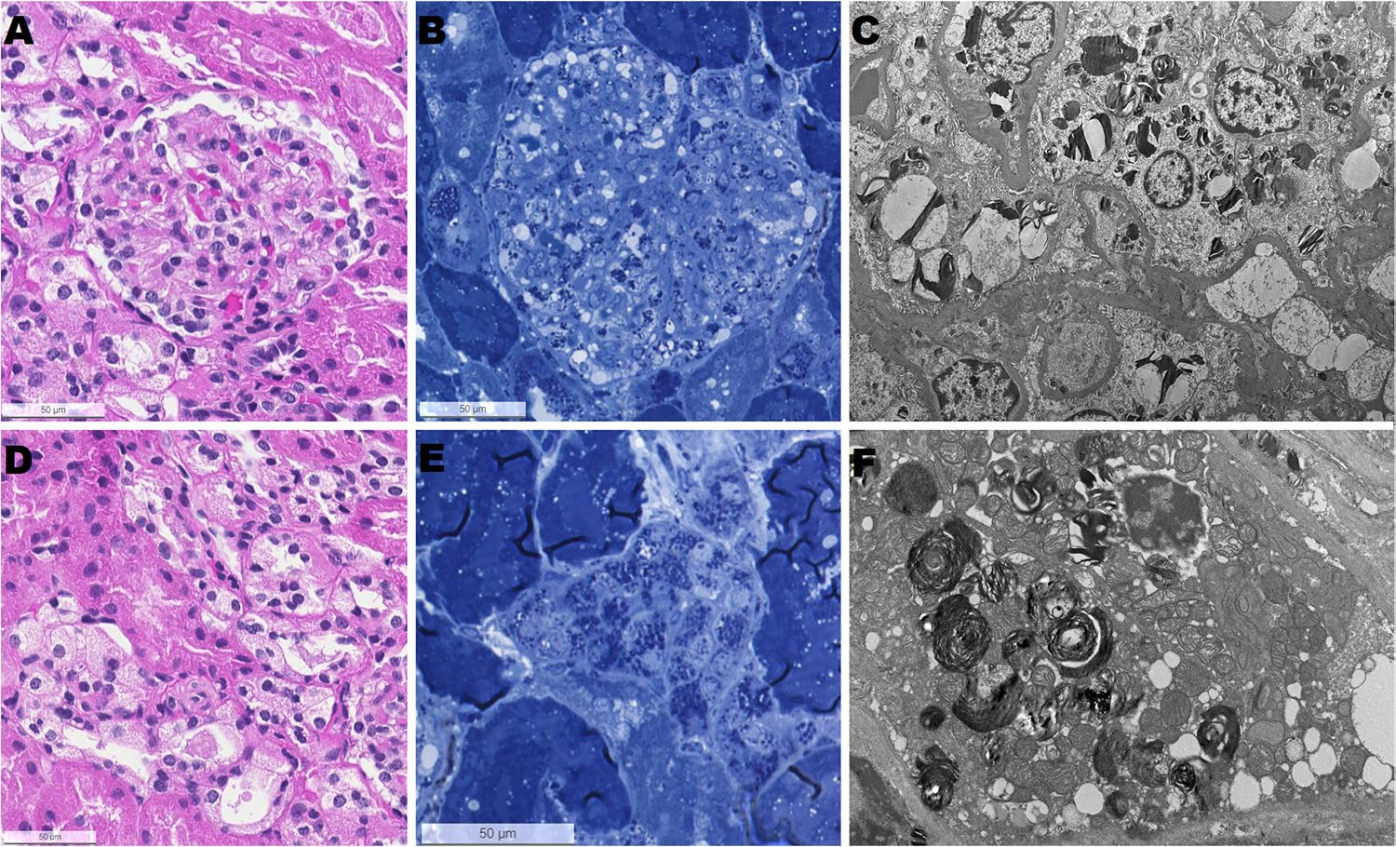
Kidney biopsy of the youngest patient (3 years old) with vacuolization of cells and glycosphingolipid deposition. **A** Glomerulus with prominent podocytes with mildly vacuolated cytoplasm (hematoxylin and eosin). **B** Glomerulus with deposits in the podocytes (methylene blue -azure). **C** Electron microscopic image of deposits in podocytes (original magnification × 700). **D** Tubules with vacuolated cells (hematoxylin and eosin). **E** Deposits in tubular cells (methylene blue -azure). **F** Electron microscopy of deposits in tubular cells (original magnification × 2000)

The main finding of our study was that vacuolization of podocytes and podocyte inclusions were found in all biopsy samples independent of genotype, clinical symptoms, age, or gender. Severe vacuolization and accumulation of inclusions in podocytes and renal tubules without laboratory abnormalities were seen in a 3-year-old, indicating that morphological changes of the kidney develop early in life and without organ-specific clinical or laboratory findings (Fig. 1). Interestingly, 80% of the patients did not have any abnormalities in their routine laboratory exams addressing kidney function, proteinuria, and albuminuria. Patients who had concurrent proteinuria or microalbuminuria showed a combination of severe vacuolization and accumulation of inclusions in podocytes, as well as segmental foot process effacement and inclusions in renal tubules in all but 3 patients. Our results rather suggest that the presence of albumin in the urine of patients with FD is a late marker, which might indicate that glomerular cells have already been irreversibly damaged. Similarly, Tøndel et al. [2] have recently demonstrated a broad spectrum of morphological changes including glomerular, tubulointerstitial, and/or vascular impairments. However, compared to the previously published results by Tøndel et al. [2], our results indicate that the kidney involvement begins even earlier.

We show that in our pediatric cohort, merely the histological evidence of kidney involvement of FD detected through biopsies leads to the initiation of ERT. This suggests that early treatment with ERT should be considered before clinical symptoms, such as proteinuria, occur, in order to prevent possibly irreversible organ damage due to Gb3 accumulation. Risk factors for CKD include low eGFR and albuminuria [17]. It can be assumed that children with classical FD, especially young male patients, develop extensive intracellular Gb3 deposits in the kidneys which triggers a cascade of cellular processes resulting in irreversible kidney damage [16]. Our findings suggest that early diagnostic kidney biopsies should be implemented independent of biochemical findings to facilitate an early indication for ERT in children with FD, taking into consideration results of several studies, which had shown small or no impact of ERT in patients with FD who had moderate to severe kidney disease [15, 31–33]. However, the effect of ERT on existing kidney damage is purely understood. Tøndel et al. [14] presented complete clearance of mesangial and glomerular endothelial cell inclusions but only 1/3 of the patients showed substantial or complete clearance of podocytes. On the other hand, Thurberg et al. [18] demonstrated moderate to severe proteinuria or reduced eGFR prior to ERT start, to be negative predictors for efficacy of ERT. A similar result was observed in patient 5b. The biopsy revealed complete clearance of glomerular endothelial cell inclusions, which may be seen as the general treatment effect [2, 14, 18]. However, despite therapy with agalsidase alfa, minimal interstitial fibrosis and glomerulosclerosis developed over time. We postulate that early kidney morphological changes can be reversed with ERT, whereas more severe changes will most probably lead to an aggravation with progressive nephropathy including interstitial fibrosis, glomerulosclerosis, and arteriopathy. Pronounced proteinuria prior to ERT might activate interstitial inflammation and fibrosis resulting in irreversible damage of the kidneys [16, 17, 21, 34–36]. Early introduction of an antiproteinuric and antiproliferative treatment is recommended in CKD to reduce progression of kidney disease [37, 38]. A combination of ERT and renin–angiotensin–aldosterone system blockade, should be implemented in FD as soon as a kidney pathology is seen in the biopsy [37, 38].

Further studies are needed to investigate the reversibility of kidney pathology and efficacy of ERT in individual FD patients. Therefore, we will further analyze kidney biopsies in our cohort after 5 years on ERT. This ongoing study will shed light on these important questions. It is essential to investigate the full effect of ERT in kidney disease of children with FD without pathological laboratory findings; the invasiveness of the diagnostic method however must be kept in mind.

Based on our findings of early morphological damages, also supporting earlier studies by Tøndol et al. [2, 7, 14] and Fogo et al. [39], ERT initiation in male classical FD should be considered soon as possible after classical FD is diagnosed. In females and children with late-onset FD, we recommend a kidney biopsy prior to ERT initiation, due to the more variable disease course and different severity [4, 22]. Corresponding to Najafian et al. [22], we assume that pre-ERT and follow-up biopsies in female and late-onset FD patients could help in the assessment of the effectiveness of different ERT dosing schemes. Meanwhile, kidney function should be monitored through regular cystatin C and plasma creatinine measurement for a more precise estimation of the GFR as well as plasma LysoGb3.

Additionally, blood pressure measurements, urine analysis for albuminuria, and kidney biopsies could potentially be used to detect early kidney damage and in turn to prevent disease progression by (1) initiating ERT and/or (2) making ERT dose adjustments.

In conclusion, our results underline and strengthen previous reports that normal standard laboratory assessments of kidney function, as well as the lack of significant classical clinical symptoms, may not exclude glomerular kidney involvement in pediatric patients with FD. Additionally, our analysis showed that kidney histological features (such as vacuolization, inclusions in podocytes and renal tubules) might be prevalently observed also at a very young age. This highlights the necessity for early treatment with ERT, i.e., before histological changes in podocytes appear, to avoid non-reversible organ damage.

Nevertheless, our study has a few limitations. Due to the rarity of FD and the single center setup, the number of individuals included in our cohort is rather small; however, our cohort represents most of the pediatric FD patients treated within Switzerland.

When considering the informative value and significance of podocyte inclusions, in particular in female FD patients, local tissue mosaicism needs to be considered and should be further investigated in future studies. Mauer et al. [40] showed an increased percentage of podocytes without Gb3 inclusions with age, suggesting survival disadvantage for FD podocytes. These findings were confirmed by Najafian et al. [41] even though robust data for females < 10 years of age is still missing.

So far only one patient had a follow-up kidney biopsy on ERT, which is mainly caused by the short period of observation. However, further analysis including more patients and multicenter cohorts are of utmost importance to allow evidence-based treatment recommendations with regards to the best age to start ERT.

Kidney biopsies, especially follow-up biopsies can help in individual decision-making and to investigate the outcome of early kidney biopsy. Incorporating detailed data on kidney morphology before and on treatment regimens, and different dosing of ERT may show that male pediatric patients with FD show the most significant benefit of ERT when treated early in life.

### Supplementary information

Below is the link to the electronic supplementary material.

Graphical abstract (PPTX 166 KB)

## Abbreviations

CKD: Chronic kidney disease
eGFR: Estimated glomerular filtration rate
EMA: European Medicines Agency
ERT: Enzyme replacement therapy
FD: Fabry disease
FDA: Food and Drug Administration
GALA: Alpha-galactosidase A
Gb3: Globotriaosylceramide
*GLA*: Galactosidase alpha gene
