# Supplementary material for: Impact of kidney biopsy on deciding when to initiate enzyme replacement therapy in children with Fabry disease
Source: Pediatr Nephrol. 2023 Jul 20;39(1):131–40. doi: 10.1007/s00467-023-06050-5 (PMC10673963; doi:10.1007/s00467-023-06050-5)
Supplement: Supplementary file 1 — Graphical abstract (PPTX 166 KB) [file 467_2023_6050_MOESM1_ESM.pptx]

## Slide 1
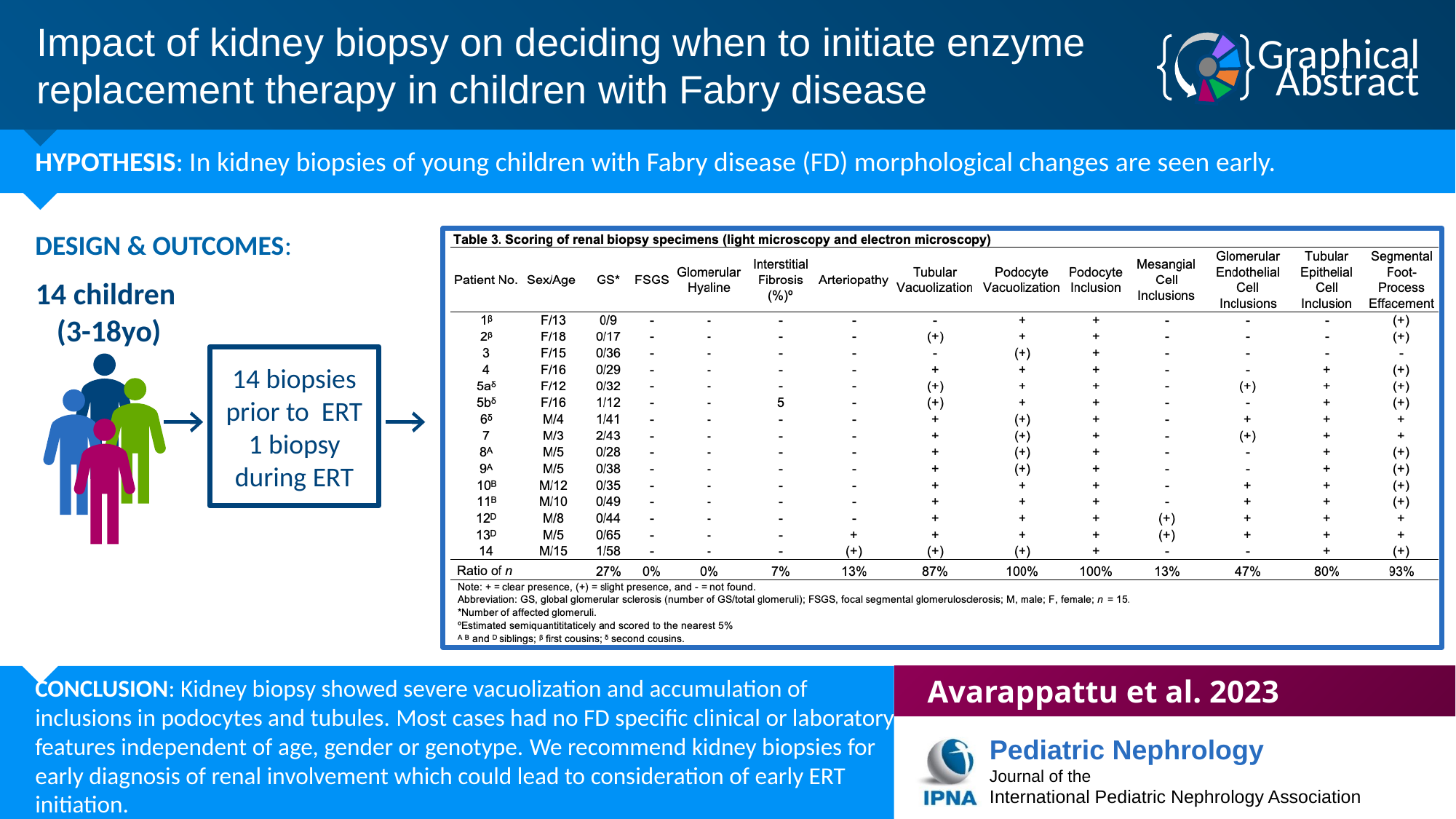

Impact of kidney biopsy on deciding when to initiate enzyme replacement therapy in children with Fabry disease
HYPOTHESIS: In kidney biopsies of young children with Fabry disease (FD) morphological changes are seen early.
DESIGN & OUTCOMES:
14 children
(3-18yo)
14 biopsies prior to ERT
1 biopsy during ERT
CONCLUSION: Kidney biopsy showed severe vacuolization and accumulation of inclusions in podocytes and tubules. Most cases had no FD specific clinical or laboratory features independent of age, gender or genotype. We recommend kidney biopsies for early diagnosis of renal involvement which could lead to consideration of early ERT initiation.
Avarappattu et al. 2023
